# A new sensory organ in “primitive” molluscs (Polyplacophora: Lepidopleurida), and its context in the nervous system of chitons

**DOI:** 10.1186/1742-9994-11-7

**Published:** 2014-01-21

**Authors:** Julia D Sigwart, Lauren H Sumner-Rooney, Enrico Schwabe, Martin Heß, Gerard P Brennan, Michael Schrödl

**Affiliations:** 1 Queen’s University Belfast, Marine Laboratory, 12-13 The Strand, Portaferry, Co. Down BT22 1PF, Northern Ireland; 2 Queen’s University Belfast, School of Biological Sciences, Lisburn Road, Belfast BT9 7BE, Northern Ireland; 3 SNSB-Zoologische Staatssammlung München, Münchhausenstraße 21, Munich 81247, Germany; 4 Ludwig-Maximilians Universität München, Biozentrum, Großhaderner Straße 2, Planegg-Martinsried 82152, Germany

**Keywords:** Chiton, Sensory biology, Osphradium, cns, Mollusca, Schwabe organ, Nervous system

## Abstract

**Introduction:**

Chitons (Polyplacophora) are molluscs considered to have a simple nervous system without cephalisation. The position of the class within Mollusca is the topic of extensive debate and neuroanatomical characters can provide new sources of phylogenetic data as well as insights into the fundamental biology of the organisms. We report a new discrete anterior sensory structure in chitons, occurring throughout Lepidopleurida, the order of living chitons that retains plesiomorphic characteristics.

**Results:**

The novel “Schwabe organ” is clearly visible on living animals as a pair of streaks of brown or purplish pigment on the roof of the pallial cavity, lateral to or partly covered by the mouth lappets. We describe the histology and ultrastructure of the anterior nervous system, including the Schwabe organ, in two lepidopleuran chitons using light and electron microscopy. The oesophageal nerve ring is greatly enlarged and displays ganglionic structure, with the neuropil surrounded by neural somata. The Schwabe organ is innervated by the lateral nerve cord, and dense bundles of nerve fibres running through the Schwabe organ epithelium are frequently surrounded by the pigment granules which characterise the organ. Basal cells projecting to the epithelial surface and cells bearing a large number of ciliary structures may be indicative of sensory function. The Schwabe organ is present in all genera within Lepidopleurida (and absent throughout Chitonida) and represents a novel anatomical synapomorphy of the clade.

**Conclusions:**

The Schwabe organ is a pigmented sensory organ, found on the ventral surface of deep-sea and shallow water chitons; although its anatomy is well understood, its function remains unknown. The anterior commissure of the chiton oesophagial nerve ring can be considered a brain. Our thorough review of the chiton central nervous system, and particularly the sensory organs of the pallial cavity, provides a context to interpret neuroanatomical homology and assess this new sense organ.

## Introduction

Chitons (Mollusca, Polyplacophora) inhabit intertidal and deep-sea marine habitats and exhibit a morphologically conserved body plan with eight articulated valves. The phylogenetic position of chitons is the focus of continuing controversy [1,2]. Where different datasets produce radically contradictory topologies within an uncontroversially monophyletic group (Mollusca), one strategy is to include novel independent sources of character data. Comparative neuroanatomy is important in phylogenetics and increasingly used for invertebrates [3]. However, there is very limited comprehensive data available for several minor molluscan classes including Polyplacophora [4]. The pallial groove in chitons, and specifically the glandular epithelium called “Schleimkrause” by Plate [5] was compared to the “wall of the shell gland of solenogastres” [6]. However, even in early literature such comparative statements have been made without substantial anatomical evidence. It is unclear how much of the documented comparative neuroanatomy of chitons has been guided by predetermined opinions about their basal position within Mollusca.

Chitons have a ventral mouth anterior to the sucking foot but no cephalised sensory structures, and are generally considered to possess a primitive nervous system which lacks true ganglia [4,7,8]. The major sensory system is the network of innervated pores (“aesthetes”) in the valves [7,9,10] and several other sensory organs have been described in the ventral pallial cavity, which runs longitudinally along either side of the foot. The nervous system consists of an oesophagial (or cerebrobuccal) nerve ring surrounding the buccal mass, typical of many protostomian taxa, which forms an anterior commissure forward of the head valve. Posteriorly, this nerve mass divides into two pairs of longitudinal nerve cords, one pair on either side of the foot (ventral, or pedal nerve cords) and the other pair entering the girdle muscle block (lateral, or pallial nerve cords) and running near to the pallial cavity. There are some exceptions to these generalities, and especially the use of the term “ganglion” has been inconsistently applied in chitons. To avoid adding to the confusion, we use the term only to describe organised structures with a central neuropil surrounded by neural somata [11], or when explicitly describing other authors’ use of the phrase. Other structures elsewhere described as “ganglia” we herein conservatively refer to as “nerves”, “nerve masses” or “neural structures”.

Living chitons are not entirely morphologically homogeneous and are clearly divided into two clades: Lepidopleurida, and Chitonida, of which the former bears the more plesiomorphic features [12]. Members of the two orders are characterised by features of the gills and shell valves [13,14]. Lepidopleurida have shells that usually lack ventral insertion plates, and in taxa where an insertion plate is present it is never slitted; they also have a gill row which grows (by serial addition of individual gills) both anteriorly and posteriorly, and in most (but not all) species the gills are restricted to the posterior part of the pallial cavity [15,16]. Species in Chitonida possess a posterior chemosensory structure on or near the gills, which has been interpreted as homologous to the osphradium in most other molluscan groups [17,18]. This structure in members of Chitonida is characterised as a raised, pigmented stripe of sensory epithelium on the roof of the pallial cavity posterior to the gill row and extending towards the anus [17,19,20]. No osphradium has been reported in Lepidopleurida, and its absence has been attributed to the posterior distribution of the gill row [21]. Many lepidopleuran chitons instead possess small lateral sense organs and potentially branchial organs, which have been suggested to fulfil the same role [17,20,22]. Finally, there have been reports of an anterior olfactory organ present in some chitons in both clades [20,23-25]. Plate [5,19,26] described many features of chiton anatomy with astonishing precision, including sensory organs in the pallial cavity and produced a wide range of impressive histological work on chiton anatomy, some of which has yet to be improved upon [17,27,28].

**Figure 1:**
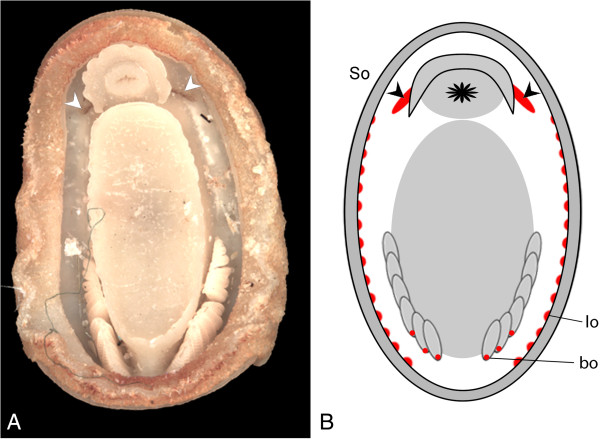
**The pallial sensory organs of Lepidopleurida. A**., Position of the Schwabe organ in *Leptochiton algesirensis*, as observed by ES; **B**., Schematic drawing indicating the sensory organs in the Lepidopleurida (generalised). The lateral organs extend through most of the pallial cavity, as shown, and the branchial organs are at the base of every gill (examples shown in 1B). So, Schwabe organs, indicated with chevrons; lo, lateral organs; bo, branchial organs.

A personal observation made by one of the authors (ES, 2008) of a specimen of *Leptochiton algesirensis* led to the identification of a structure not previously described in the historical literature: an elongated pair of patches of brown or purplish pigment stretching posterior from beneath the mouth lappets towards the start of the foot, and extending laterally on either side of the mouth (Figure 1) [16,29]. This structure is clearly visible to the naked eye. Our work has determined the ultrastructure of this large and complex tissue region, which we refer to as the “Schwabe organ”, after its discoverer, and here the results are examined in the context of other historical studies of chiton sensory anatomy.

## Results

**Figure 2:**
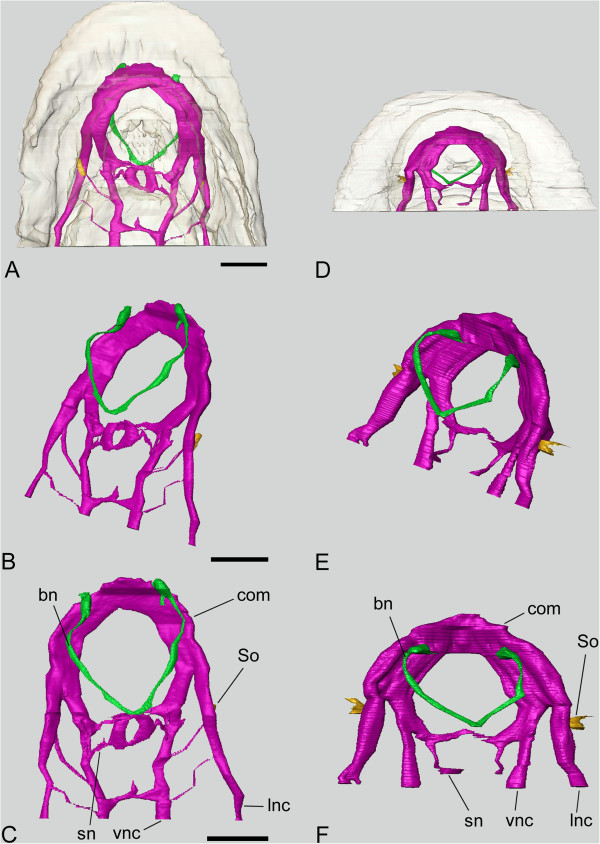
**Tomographic models of the anterior nervous system in *L. asellus *(A-C) and *L. rugatus *(D-F). A**., **D**., Ventral view with outline of body shown; **B**., **E**., Angled dorsal view of nervous system and Schwabe organs; **C**., **F**., Dorsal view of nervous system. Pink, nerve tissue; green, buccal nerves; yellow, Schwabe organs. bn, buccal nerves; com, anterior commissure; lnc, lateral nerve cord; So, Schwabe organ; sn, subradular nerves; vnc, ventral nerve cord. Scale bar 500 μm.

The Schwabe organ lies slightly posterior to the mouth within the pallial cavity. It is visible as a stripe of dark pigment in the surface epithelium (Figure 1A). This pigment is also distributed in cell bodies beneath the basal lamina (see TEM, below). The Schwabe organ region is innervated by the lateral nerve cord and is connected to it slightly posterior to the point where the nerve cords split from the anterior commissure (Figure 2).

### Anterior nervous system of *Leptochiton* spp

**Figure 3:**
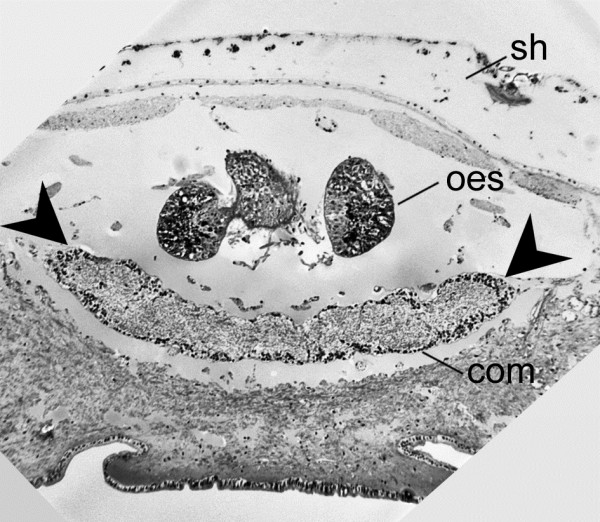
**The anterior commissure in *L. rugatus*, showing internal fibre region and peripheral cell bodies.** The commissure (brain; *com*) is the anterior expansion of the oesophageal nerve ring; a cross section shown here extends between the chevrons, with internal neuropil surrounded by dark nuclei. Com, anterior commissure, also bracketed with chevrons; oes, supraoesophagial pouch epithelium; sh, shell.

Tomographic models of the anterior nervous system in *Leptochiton asellus* and *Leptochiton rugatus* give an accurate representation of anterior chiton neuroanatomy (Figure 2, Additional files 1 and 2). The anterior commissure is large, oval in cross-section and flexed upwards distally (Figures 2, 3). It encircles the mouth and splits equally into two pairs of major nerve cords posterior to the mouth, and anterior to the subradular nerves (paired central neural structures ventral to the radula bolster). These are the lateral (or pallial; the distal pair) and the ventral (or pedal; proximate pair) nerve cords. The buccal nerves are two large discrete structures situated dorsally within the body at the posterior margin of the oesophageal nerve ring, and are conjoined at a point dorsal and slightly anterior to the subradular nerves. The subradular nerves form a bridge (commissure) between the two ventral nerve cords. There is a second substantial bridge posterior to this which is visible in the model of *L. asellus* (Figure 2A–C). Posterior to this commissure, the nerve cords maintain a consistent diameter through the rest of the body. The model of *L. asellus* is comparatively more extensive than that of *L. rugatus* and also captures smaller commissures connecting the ventral and lateral cords on each side at regular intervals.

The anterior commissure is substantially more massive in comparison with the remainder of the suboesophageal ring and the lateral and ventral nerve cords (Figure 2). The surface thickness on the dorsal face is on average over 220% wider than the lateral nerve cord in *L. asellus* (Figure 2C)*.* The anterior commissure in *L. asellus* is a semicircle of roughly uniform thickness, but in *L. rugatus* it is around 65% wider at the lateral margin prior to the division of the major nerve cords (Figure 2F).

**Figure 4:**
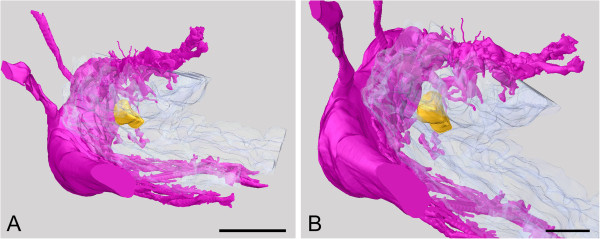
**Tomographic model of the Schwabe organ in *L. asellus.*** The anterior end is in the foreground, and the dorsal side at bottom. **A**., View of the entire tomographic model (scale bar 50 μm); **B**., Innervation of the Schwabe organ (scale bar 25 μm). Pink, nerve tissue; white, epithelium; yellow, Schwabe organ.

On each side of the body, the lateral nerve cord lies very close to the Schwabe organ in both species examined, curving around the region and thus giving a high level of contact between the nerve cord and the pigmented region (Figures 2, 4). This is confirmed by the higher-resolution model of the pigmented region in *L. asellus*, which shows that there are many smaller nerves separating from the lateral nerve cord and entering the epithelium in the region of the Schwabe organ, including an epithelial projection (Figure 4).

### Schwabe organ

**Figure 5:**
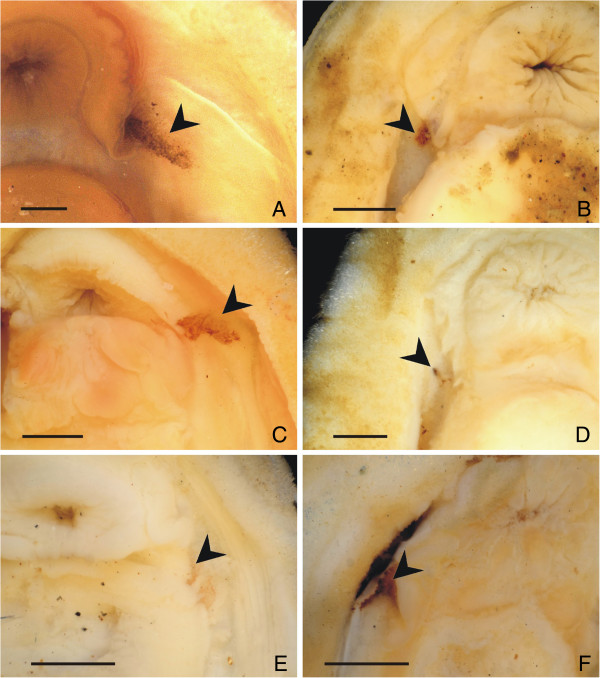
**Anterior ventral side in various Lepidopleurida, to illuminate the variability of the Schwabe sense organ.** All scale bars 1 mm. **A**., *Leptochiton asellus* (ZSM Mol 20130056), Northern Ireland, Strangford Lough, intertidal; **B**., *L. belknapi* (ZSM Mol 20041461) Chile, off Concepción, 900 m; **C**., *Parachiton acuminatus* (ZSM Mol 20052008) Samoa, Savaii Island, Lepela, 0.5-3 m; **D**., *Deshayesiella curvata* (ZSM Mol 20100176) Russia, Vostok Bay; **E**., *Ferreiraella plana* (MNHN 30986) Vanuatu, off NE Tutuba Island, 759–985 m; **F**., *Oldroydia percrassa* (ZSM Mol 20040612) Mexico, off Arbolito, 60–75 m.

The Schwabe organ is present in all examined species of Lepidopleurida (Figure 5, Table 1), and we infer it is present in all members of the order. The externally visible morphology varies from a small concentrated dot of pigment in some taxa, to a stripe of pigment extending posteriorly to the front part of the foot in others.

**Table 1:** Taxonomic arrangement of the polyplacophoran order Lepidopleurida, and genera that have been formerly referred to Lepidopleurida, and occurrence of the Schwabe organ

**Suborder**	**Family**	**Genus**	**Schwabe organ**
Lepidopleurina	Ferreiraellidae	*Ferreiraella*	present
	Hanleyidae	*Hanleya*	present
	Leptochitonidae	*Hanleyella*	present
		*Lepidopleurus*	present*
		*Leptochiton*	present
		*Parachiton*	present
		*Pilsbryella*	unknown
	Nierstraszellidae	*Nierstraszella*	present
	Protochitonidae	*Deshayesiella*	present
		*Oldroydia*	present
Acanthochitonina	Choriplacidae (C)	*Choriplax* (C)	absent
	Hemiarthridae	*Hemiarthrum* (C)	absent
		*Weedingia* (C)	unknown**

The authors have visually examined specimens in these genera for external pigment patches associated with the sensory structure described. Genera that have been suggested as members of Chitonida (Sirenko, 2006) but formerly referred to Lepidopleurida are noted (C).

*Small patches, very close to the head, where they occur in deep grooves.

**Investigated in one specimen and not observed in preserved material, but cannot be definitively excluded (Sigwart et al., 2013).

**Figure 6:**
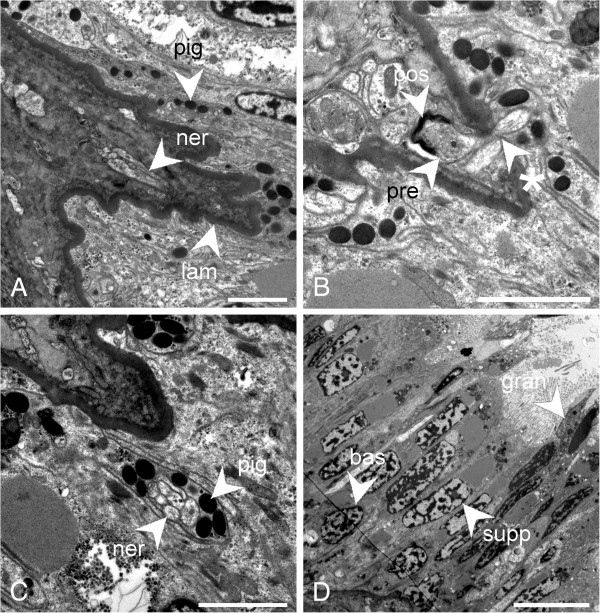
**The ultrastructure of the Schwabe organ in *Leptochiton asellus*. A**., Nerve projecting towards the base of an epithelial projection; **B**., Nerves leaving the epithelium form a synapse in the mesoderm; **C**., Nerve bundle surrounded by pigment granules in the epithelium; **D**., An overview of the sensory epithelium. Abbreviations: bas, basal cell; cil, cilia; gran, granular cell; lam, basal lamina; ner, nerve; pig, pigment granule; pre, putative presynaptic vesicles; pos, putative post-synaptic density; sup, supporting cell; asterisk, nerve penetrating the basal lamina. Scale bars: A., B., C. = 2 μm; D. = 10 μm.

TEM visualisation of cell type and ultrastructure were conducted on specimens of *L. asellus*. A large nerve (diameter up to 30 μm) separates from the lateral nerve cord and branches as it approaches the surface epithelium to give numerous fine nerve bundles (Figure 6A). These bundles, containing up to 15 axons each, then penetrate the basal lamina and innervate the pigmented epithelium (bundle diameter up to 2 μm, Figure 6B). In some cases, they appear to form what could be synapses with nerves in the deeper tissues, with a concentration of presynaptic vesicles found in the nerve coming from the epithelium and putative postsynaptic density in the mesoderm (Figure 6B). The nerves in the epithelium are frequently observed to be directly surrounded by cells containing pigment granules similar to those identified by Leise and Cloney [30] (Figure 6C). The epithelium comprises a single layer of cells, the surfaces of which bear microvilli and some of which also bear cilia. Cells can be closely packed, sometimes forming two rows, or an appearance of a double-layer of cells (pseudostratification). Pigment granules are numerous, but generally basally located (Figure 6A). We infer that membrane-enveloped pigment granules may be traversing outward from the mesoderm, across the basal lamina and into the exterior epithelium (pigment clusters shown in the bottom half, epithelium, leading away from the basal lamina, Figure 6A–C).

**Figure 7:**
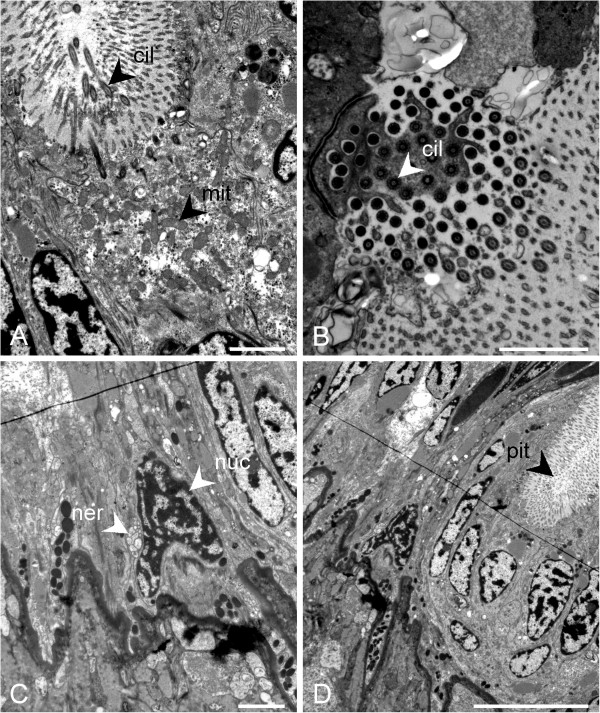
**The ultrastructure of the Schwabe organ in *Leptochiton asellus *(continued). A**., Ciliary cell; **B**., Cross-section through the cilia originating in one ciliary cell; **C**., Nerve bundle associated to the nucleus of a basal cell; **D**., Invagination of the epithelium creates a pit. Abbreviations: cil, cilia; mit, mitochondrion; ner, nerve; nuc, nucleus; pit, epithelial pit. Scale bars: A., B., C. = 2 μm; D. = 10 μm.

There are four major cell types present in the epithelium. Supporting cells are the most common, and are around 30 μm in height with distally located oval nuclei, apical microvilli and basally located pigment granules. At the apical side of the nucleus they also have a large vesicle-like structure with low electron density, and apical to this, smaller vesicles with a granular appearance (Figure 6D). At the medial edge of the epithelium, some cells also possess a large dark vesicle at the apical side of the cell. The second type is a multiciliary cell, which is present in relatively small numbers. These cells are around 20 μm in height and contain a large number of mitochondria, a round nucleus and many shallow-rooted cilia (over 80 in some cases, Figure 7A, B). Between the supporting cells are basal cells, with a cell body around 10 μm in height, projecting towards the surface of the epithelium, and round, basally located nuclei and pigment granules, giving the epithelium a pseudostratified appearance (Figure 6D). These are often in direct contact with the aforementioned bundles of nerve fibres, which are sometimes directly adjacent to the nucleus (Figure 7C). Finally, there are several very thin, elongated cells around 35 μm in height and 2 μm wide which contain one large electron-dense vesicle and many smaller vesicles, giving them a granular appearance (Figure 6D). At several sites, an invagination of the epithelium is visible (Figure 7D). This is 6 μm across, and is caused by the presence of cells resembling support cells, but only 10 μm in height, thus creating a dip or “pit” in the apical surface of the epithelium. Additionally, a projection of the epithelium from the area connecting the mouth lappets to the pallial cavity was also observed. This projection is large, around 75 μm in diameter and was observed in all specimens. It is also directly innervated by a large bundle of nerve fibres (Figure 6A). We were unable to conclusively identify this structure in the surface epithelium in SEM studies of the anterior pallial cavity.

By comparison, the epithelium anterior of the Schwabe organ region, i.e. anterior of the mouth in the pallial cavity roof, is distinctly different. The pigment is absent (both visually from living specimens, and confirmed by histology and TEM); although nerves are also present and penetrating the basal lamina, the epithelium is thinner, around 20 μm instead of 40 μm thick; and the major cell layer is composed of cells that have nuclei situated more basally, with a well-developed basal labyrinth and the cells are interlocking rather than columnar.

Observations of the external surface in *L. asellus* via SEM revealed an area of slightly raised epithelium containing several large pores around 7 μm in diameter which could correspond to the pits observed in the TEM (above). Similar pores are found throughout the pallial cavity, but those located more posteriorly are slightly smaller in diameter.

### Other sensory structures

**Figure 8:**
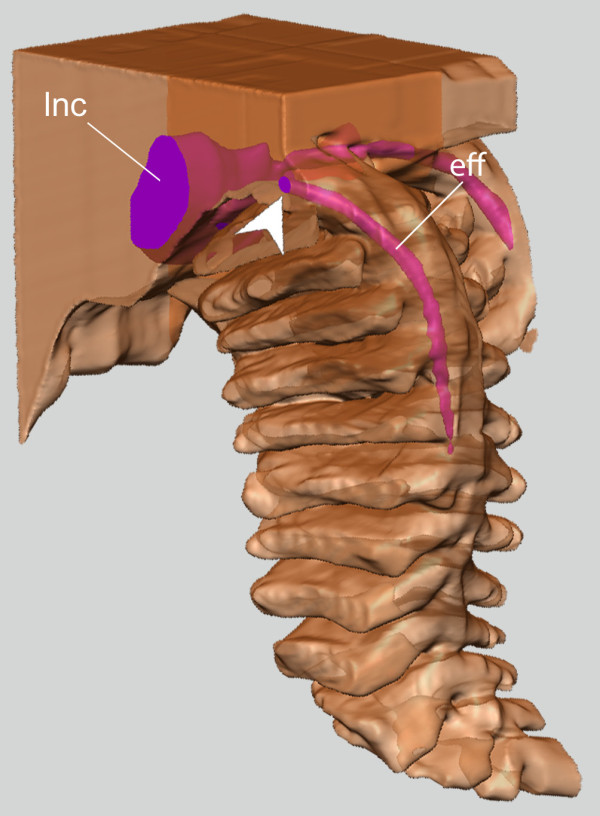
**The position of the “branchial sense organ” on the gill in *Leptochiton rugatus*.** The lateral nerve cord is visible through the pallial epithelium, as is the efferent nerve penetrating the individual gill. Chevron: branchial sense organ; lnc, lateral nerve cord; eff; efferent nerve.

The lateral nerve cord approaches the surface of the epithelium within individual gills in *L. rugatus* and may form a small ridge at the base of the gill previously interpreted as a branchial sense organ (Figure 8). In all the species examined we found no evidence of external surface pigment associated with this region.

**Figure 9:**
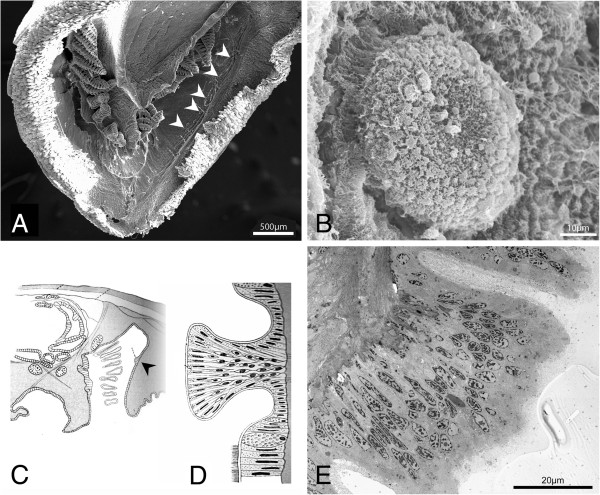
**The ultrastructure of the lateral sense organs. A**., Scanning electron microscope (SEM) image showing the position of the lateral sense organs (chevrons) in the posterior pallial cavity of *Leptochiton asellus*; **B**., SEM image showing one lateral sense organ in *L. asellus*; **C**., Diagram of a cross-section through the body of *Hanleya hanleyi*, showing the presence of the lateral organ in the gill row, indicated by chevron, from Plate, 1901, pl. 5, Figure one hundred ninety-seven; **D**., Diagram of a cross-section through a lateral sense organ in *Lepidopleurus cajetanus*, from Plate, 1901, pl. 5, Figure two hundred twelve; **E**., TEM image showing a section through a lateral sense organ in *L. asellus*.

The lateral organs are present in high numbers and throughout the pallial cavity in both *L. asellus* and *L. rugatus*, extending from the posterior side of the mouth to a point level with the anus. Lateral sense organs are ellipsoid mounds around 30 μm across and extending around 20 μm above the epithelium. They lie along a fairly consistent axis on the lateral wall of the pallial cavity, around 70 μm from the edge of the girdle (Figure 9A–C). In the larger of two specimens of *L. asellus* (length 7.3 mm), 23 pairs of lateral sense organs were counted, and 10 pairs were found in the smaller specimen (5 mm). In a specimen of *L. rugatus*, 14 pairs were counted from serial sections. Observations from semi-thin sections could not reliably differentiate candidate lateral organs in the epithelium in the gill row, which is relatively thicker than the epithelium in the central part of the pallial cavity.

A lateral organ is formed of stacks of cells approximately 40 μm thick (Figure 9D, E). These can be discrete pads raised above the surrounding epithelium (Figure 9D) or a constriction around bundles of cells. The cells do not form sequential layers but have interdigitating processes that give the appearance of stacked nuclei (Figure 9D, E). There are four types of cells associated with the lateral organ of *L. asellus* (Figure 9E) which correspond to the descriptions from *Lepidopleurus cajetanus*[17]: supporting cells with distal oval nuclei and microvilli; mucous cells at the margin or immediately outside the lateral organ (*cf.* Figure 9D); small cells with round basal nuclei and fine longitudinal processes that reach the surface, and putatively sensory ciliary cells on the surface layer which are connected to intraepithelial nerve fibres.

## Discussion

### The Schwabe organ

The anatomical features we have described above strongly support the characterisation of the Schwabe organ as a sensory structure. The close association to the lateral nerve cord and the dense innervation of the surrounding epithelium both indicate the importance of nervous activity in the area (Figures 2, 4). Crucially, nerves cross the basal lamina and may form synapses with nerves in the mesoderm (Figure 6B), and a concentration of presynaptic vesicles is found in the nerve leaving the epithelium, thus implying the transfer of information from the epithelium inwards. The histological features described above could also be interpreted to suggest the presence of glandular tissue in the region of the Schwabe organ. Although we consider this less likely than a sensory role, the two functions are not necessarily mutually exclusive. The Schwabe organ shares many features with other sensory epithelia described in chitons, including the presence of supporting, granular and cilia-bearing cells alongside basal cells carrying epithelial projections [17] and it differs from the epithelium found in other areas of the pallial cavity. Numerous nerve bundles found throughout this epithelium are clearly associated to the pigment. Its position on either side of the mouth may also indicate sensory function. Thus, the Schwabe organ is at least as well defined in terms of position and histology as any other sensory structure in the pallial cavity of chitons (see below).

The specific functions or roles of the sensory structures within the pallial cavity, including the Schwabe organ, remain unknown. Pigmentation appears in many molluscan neural structures [17,18,30,31], but pigmented sensory structures are often associated with photosensitivity [30,32]. The Schwabe organ is ventral, within the pallial cavity, and actually is further hidden in life position by the mouth lappet or hood. The Schwabe organ is apparently present in all members of the Lepidopleurida, which is a predominantly deep sea clade [12]. It therefore would seem improbable that the Schwabe organ, or indeed any of the pigmented sensory epithelia in the pallial cavity of any chitons, is photosensitive.

Speculation about the function of sensory organs in the pallial cavity primarily focus on chemosensitivity [19], preventing sediment overloading [20], or synchronisation of broadcast spawning events [17]. Yonge [20] criticised the hypothesis that the posterior osphradium functioned to test water quality, as it lies behind most of the gills and the prevailing water current runs anterior to posterior through the pallial cavity. The Schwabe organ is clearly anterior to the gill row in all species but chitons can bring water into the pallial cavity under the girdle along the whole length of the body [20].

We did not observe localised concentrations of long, short-rooted cilia on the Schwabe organ that would be expected with chemosensory structures, but this cannot and should not be excluded. Bundles of cilia were observed here, but similar cilia are found throughout the pallial cavity of other non-lepidopleuran chitons [33] and may be primarily used in generating respiratory water currents. All of these ideas provide discrete and testable hypotheses, however they do not offer any explanation for the absence of the Schwabe organ in the Chitonida. The Schwabe organ is an apparent synapomorphy of Lepidopleurida. We consider previous reports of the “anterior sensory organs” *sensu* von Knorre [25] to be artefacts or certainly not the same structure (see below). Within Lepidopleurida, the presence and position of the pigmented region of the Schwabe organ is highly consistent, although its shape and size are variable (Figure 5).

### The nervous system of chitons

The central nervous system (cns) of chitons has been commonly considered to lack any true ganglia; for example Mizzarro-Wimmer and Salvini-Plawen [34] described the oesophageal nerve ring as “ganglionic”, but emphasised that no ganglia are developed. Other authors recognised subradular ganglia and/or buccal ganglia [4,28], but stated that polyplacophorans lack a distinct ganglionic brain [4].

Tetraneural nerves are either considered as having ganglia or as medullary cords (having a central neuropil with somata distributed along the length of the cord) [11]. Shigeno et al. [35] considered the lateral and ventral nerve cords of the aplacophoran *Chaetoderma japonicum* to be “ganglionated”, identified a brain (composed of cerebral ganglia) including lobate structures, and compared them to the anterior commissure in a chiton (Chitonida: *Lepidochitona* sp.). Within a traditional morphological (testarian) evolutionary framework, development of neural structures would be expected to increase in complexity from the supposedly plesiomorphic vermiform aplacophorans, becoming more organised in chitons, and moving towards true ganglia in “higher” conchiferan groups such as gastropods and cephalopods. However, progressive evolution from simple worm-like to shell-bearing molluscs is not consistent with molecular phylogenetics [1,2]. Although there is ample evidence of vermification in the polyplacophoran stem lineage [36,37] this is also not conclusive ‘‘proof’’ of the plesiomorphic nature of chitons [2]. The balance of evidence for molluscan evolution suggests repeated secondary reduction of shell and other organ systems including the cns (for example in bivalves). Data on the chiton cns and other minor classes are so far scarce [4], and we consider that early observations on the chiton anterior commissure may have been interpreted and illustrated in a concept-driven and potentially misleading way.

There seems to be a gradual rather than discrete transition between medullary cords and true ganglia in molluscs. Our results show that the circumoesophageal ring is structured like a ganglion, with internal fibre region (medulla) and peripheral cell bodies (cortex, Figure 3). This is in accordance with immunocytochemical results by Faller et al. [4]. However, there are also several subtle constrictions around the nerve ring (Figures 2, 3), thus the anterior commissure rather resembles a fused sequence of ganglia, without detectable borders, rather than simply a medullary cord, and may well be a brain as defined by Richter et al. [11]. Our 3D models illustrate the significant enlargement in the anterior commissure compared to the rest of the anterior nervous system (Figure 2), which has not been accurately represented in much of the previous literature [4,24,38,39]. For example, in adult *Leptochiton* spp. the anterior commissure in the two species we studied is around 200% wider than the lateral nerve cord and 220% wider than the ventral nerve cord, immunohistochemical visualisation of the same region in juvenile *Lepidochitona cinerea* (Chitonida) show these figures to be just 75% and 12.5% respectively ([4], fig. 2.1). The two studies used different techniques, and FMRFamide staining detects only a subpopulation of the cells in the molluscan nervous system [4,40]. The presence of non-FMRFamide staining cells, or the dense aggregation of nuclei observed in the distal margin of the anterior commissure, may account for around one-third of its cross sectional thickness. The substantial variation in the relative inflation of the anterior commissure could be more than artefactual, and adults may have a relatively larger anterior neural mass than newly-settled juveniles, or *Leptochiton* spp. Also, members of Lepidopleurida may simply have a larger neural mass than Chitonida.

There are notable distinctions between *L. asellus* and *L. rugatus*, such as the shape and position of the buccal neural structures (Figure 2). Most aspects of polyplacophoran nervous systems, including these, have only been described in detail from a small number of species. Immunoreactive characters of the nervous system have been studied in *Leptochiton asellus* (one of our study species) [8], and *Lepidochitona cinerea* and *Acanthochitona crinita*[4]. Broader taxonomic sampling is needed to establish morphoanatomical characters suitable for phylogenetic inference. Our results, particularly the Schwabe organ as a feature of Lepidopleurida, provide a framework for identifying relevant characters from chiton nervous systems.

Chiton sensory structures include the shell pores or aesthetes, which are known to be innervated [10] and may have a primarily chemosensory [19,41] or tactile [26] function, but in several cases they are secondarily adapted as photosensitive eye spots including as lensed and image-forming eyes [10,42-45]. There are also possibly photoreceptive sensory elements [46,47] and mechanosensory structures on the girdle [48]. A number of sensory features are also found in the pallial cavity, which generally contains extensive sensory epithelium and glandular tracts with contain neurosensory cells [49]. Discrete sensory organs within the pallial cavity appear to have species-specific features [49] and it is these structures which are discussed below.

### A history of sensory organs in the pallial cavity of chitons

**Figure 10:**
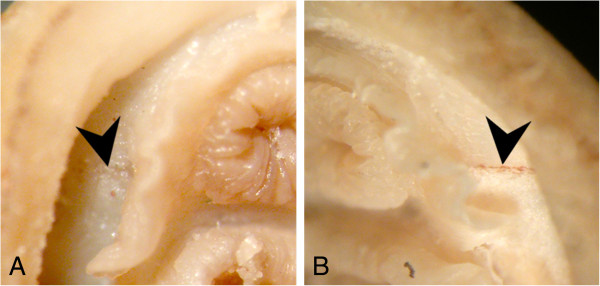
**Preservation artefacts affect the visibility of the Schwabe organ in fixed specimens. A**., Specimen of *Leptochiton boucheti*, preserved in 95% ethanol and showing bleaching; **B**., A second specimen of *L. boucheti*, fixed in formalin and subsequently stored in 70% ethanol, with pigmentation intact. Specimens from the collection of the Muséum National d’Histoire Naturelle, Paris (MNHN). Chevron: Schwabe organ.

It seems unusual that a feature as visually prominent as the Schwabe organ should remain undiscovered for more than 100 years of anatomical study, given the comprehensive histological descriptions provided by early authors e.g. [5,19,26]. We have observed that ethanol preservation causes bleaching of the pigment to the point where it is almost invisible (Figure 10). This effect is less pronounced in material initially fixed in formalin or glutaraldehyde, but storage in alcohol can still cause bleaching. In dying or damaged animals, the pigmentation in the epidermis fades very rapidly. This also occurs when the living tissue in the Schwabe organ region is excised. It is therefore perhaps not so strange that many anatomists have overlooked this pigmentation on preserved specimens, or assumed it was surface debris or dirt.

To elucidate the role or function of the Schwabe organ we have reviewed the literature on relevant sensory anatomy (Table 2), focussing on four previously described putative sensory structures within the pallial cavity of chitons. There is a general association of pigment with sensory epithelia in chitons and other molluscs [18]. But positional homology is at least as important as the presence or absence of pigment, especially given the transient preservation of pigmentation in the Schwabe organ region.

**Table 2:** Presence or absence of different pallial sensory organs in the Polyplacophora

**Taxa**	**lo**	**O**	**bo**	**Sources**	**Notes**
**Leptochitonidae**					
*Lepidopleurus cajetanus*	+	-	+	Plate (1899); Yonge (1939); Haszprunar (1981, 1987)	
*Leptochiton asellus*	+	-	+	Plate (1899); this study	Yonge (1939) failed to find the lateral sense organs in this species
*Leptochiton medinae*	+	-	+	Plate (1899)	
*Leptochiton rugatus*	+	-	+	This study	
**Hanleyidae**					
*Hanleya hanleyi*	+	-	+	Burne (1895); Plate (1899)	Partly as *H. abyssorum*; “At the front [of the half shell groove] are found discrete bumps or strips of higher, but not as sharply defined, epithelium than the surrounding epithelium, which is possibly also sensory. (Plate, 1899, p. 78)
**Callochitonidae**					
*Callochiton septemvalvis*		–		Plate (1899)	As *C. laevis*
**Ischnochitonidae**					
*Ischnochiton bouryi*		+	“+”	von Knorre (1925)	As *I. aequigranulatus*; the author does not explicitly describe a branchial sense organ, but mentions a lateral respiratory epithelium
*Ischnochiton rissoi*		+		Haszprunar (1987)	
*Ischnochiton stramineus*		+		Plate (1899)	As *I. imitator*
*Stenoplax alata*		+		Plate (1901); von Knorre (1925)	Partly as *I. herdmani*
*Stenoplax conspicua*		+		Plate (1901)	
**Callistoplacidae**					
*Calloplax vivipara*		–		Plate (1899)	
**Chaetopleuridae**					
*Chaetopleura peruviana*		+		Plate (1899)	
**Chitonidae**					
*Chiton corallinus*		+		Blumrich (1891)	Partly as *C. laevis* (see Thiele 1895a)
*Chiton cumingsii*		+		Plate (1899)	
*Chiton olivaceus*		+		Blumrich (1891); Plate (1899); Haszprunar (1981, 1987)	Partly as *C. siculus*
*Chiton* sp.		+		Thiele (1890)	
*Tonicia atrata*		+		Plate (1901)	As *T. fastigiata*
*Acanthopleura echinata*		+		Plate (1901)	
*Acanthopleura gemmata*		+		Kamardin (1988, 1989)	Most probably *A. brevispinosa*.
*Enoplochiton niger*		+		Plate (1901)	
**Tonicellidae**					
*Lepidochitona caprearum*		+		Blumrich (1891); Haszprunar (1987)	Partly as *C. polii*
*Lepidochitona cinerea*		+	+	Pelseneer (1898); von Knorre (1925); Yonge (1939); Haszprunar (1987)	Partly as *Boreochiton marginatus*; von Knorre (1925) mentioned the branchial sense organ.
*Boreochiton rubra*		+		Plate (1899)	
*Tonicella marmorea*		+		Plate (1899); Yonge (1939)	
**Mopaliidae**					
*Nuttallochiton martiali*		+*		Plate (1898; 1899)	As *N. hyadesi*
**Acanthochitonidae**					
*Acanthochitona crinita*		+		Yonge (1939)	
*Acanthochitona fascicularis*		+	+**	Blumrich (1891); Plate (1901); Haszprunar (1981, 1987)	Partly as *A. communis*
*Cryptoconchus porosus*		+		Plate (1901)	
*Notoplax violacea*		+		Plate (1901)	
**Cryptoplacidae**					
*Cryptoplax oculatus*		+		Plate (1901)	

The marking for the osphradium indicates whether or not it was found posteriorly next to the anus. Not included is the anterior olfactory organ observed by several authors: in *Lepidopleurus cajetanus* and *“Chiton” laevis* (Blumrich, 1891), *Ischnochiton herdmani* (*Stenoplax alata*) and *I. aequigranulatus* (*I. bouryi*) (von Knorre 1925), *Lepidochitona cinereus* but not *Leptochiton asellus*, (Yonge 1939), *Callochiton septemvalvis* (Thiele, 1985), and*“Boreochiton* (Pelseneer 1898).

*Plate (1899, p. 163) explained that osphradia are missing but corrected in Plate (1901, p. 427) bo – branchial sense organ; O – osphradium; lo – lateral sense organ.

**Referred to as “osphradium” by Blumrich (1891) but see detailed review in the main text.

There are several sensory features in the pallial cavity, all of which are innervated by the lateral nerve cords ([49], p. 99). Interestingly, if the lateral (pallial) nerve cord is cut, the gills may coordinate locally, not relying on communication with the anterior commissure ([46], p. 256). The whole of the pallial cavity may be lined with innervated glandular epithelia and contains differentiated mucous tracts that aid transport of sediment material. The term *Schleimkrausen* was used by several early authors to describe both the general nature of the pallial epithelium and specific regions of interest within it [49]. In fact, Nierstrasz [50] noted that the discrete sensory structures within the pallial cavity had been interpreted variously by different authors.

### Larval eyes

While not actually a sensory organ within the pallial cavity, larval eyes on the chiton trochophore [51] migrate during development to a dorsal position which is otherwise similar to that of the Schwabe organ in adult members of the Lepidopleurida [39]. The larval eyes are also innervated by the lateral nerve cord [52] as are pallial sensory structures in the adult animals. Kowalevsky [52] first described the larval eye as a deposition of pigment around a clear central component, situated just above the lateral nerve cords. However, Heath [39] described them as a dorsal feature. Rosen et al. [53] and Bartolomaeus [54] considered the larval ocelli to be homologous to cephalic eyes in other molluscs, but they are clearly posterior to the prototroch and not directly connected to the potentially cephalic anterior commissure [51]. The developmental fate of nerves associated to the larval eye after metamorphosis and homology with adult features has not been investigated in detail.

### Lateral organs

The lateral sense organs, small sensory buttons in the distal wall of the pallial cavity, were first discovered and identified as such by Thiele [55], who observed approximately 35 of them in the mantle cavity of *Lepidopleurus cajetanus* but did not find any in Chitonida. He compared them with the lateral sense organ of *Haliotis*, concluded that they were homologous, and on this basis proposed their sensory function [55].

Plate ([19], p. 430) concurred with Thiele [55] in that he could not find the lateral sense organ in any non-lepidopleuridan genera. He also found the same structure in *Leptochiton medinae* (12 per side) and *Hanleya hanleyi* (not counted), and concluded they are indeed restricted to lepidopleurans (Table 2). Plate [19] further described the structure of the lateral organs, stating that the innervation originates from the mantle and connects to the “*Seitenmarkstraenge*” (lateral nerve cord). One schematic diagram shows their distribution at approximately regular intervals and extending posterior to the gonopore and nephropore, and a second illustration confirms that the lateral sense organs extend into the gill row (Figure 9C, D). Plate [19] suggested that these sensory organs should be considered olfactory and might function in testing or tasting water in the pallial cavity.

Yonge [20] summarised the previous reports of Plate and Thiele and suggested that the epithelium is the same type as other sense organs in the pallial cavity (branchial, osphradial, and anterior; see below). These features remain known from only four species including the two described herein (Table 3). Haszprunar [17] described the histology of lateral organs in *Lepidopleurus cajetanus* and defined them as being 20 μm higher than the surrounding epithelium, anterior and external to the gill row, and thus in the inhalant chamber. The lateral organ contains four types of cells: columnar epithelial cells, mucous cells, and two types of ciliated cells, one with a fine process penetrating the main epithelial columnae, and the other “at the edge” of the epithelium and bearing cilia. These were originally reported as “paddle-cilia”, which are in fact an artefact of fixation [56]–[58]. Our histological results concur with Haszprunar’s ([17], p. 41) description; however, we did not observe cilia, and the mucous cells he described as being within the lateral organ appear to be mucosecretory cells actually in the groove surrounding the lateral organ pad (Figure 9D). The region illustrated in that publication did not include the central part of the lateral organ (Figure 9D, E) but rather the edge of one lateral organ ([17], fig. 15). This sensory epithelium, at the centre of the lateral organ, is the same thickness as other sensory epithelia described in the Schwabe organ (herein) and for the osphradium [17]. The pallial cavity epithelium is variable in thickness and in areas where there is thickening (such as proximate to the gills) it is impossible to distinguish minor folds from specific lateral organs in semi-thin sections. The lateral organs are distinctly visible under SEM (Figure 9A, B) but this may have led to under-counting of the lateral organs in historical reports [19,55]; we consider our reported counts for *L. asellus* to be the most accurate for any species so far examined (Table 2, Table 3).

**Table 3:** Number and distribution of lateral sense organs in members of the Lepidopleurida described in studies to date

**Species**	**Lateral sense organs (per side)**	**Anatomical distribution**	**Source**
*Leptochiton medinae*	12	Posterior of the nephropore	Plate (1901)
*Lepidopleurus cajetanus*	Up to 35 (Thiele), fewer in a smaller specimen (Plate)	Posterior of the nephropore	Thiele (1895); Plate (1901)
*Leptochiton asellus*	10 (specimen 5 mm), 23 (specimen 7.33 mm)	From end of foot (at gill 3 in larger specimen) to just posterior of the mouth.	This study
*Leptochiton rugatus*	At least 14	From gill 7, and possibly further posterior, to just posterior of the mouth.	This study

(n.b. Polyplacophoran gills are counted with 1 being the most posterior gill in a specimen).

The lateral organs are relatively easily defined and have been described in several taxa. Yet the history of description of even this structure illustrates some of the challenges, which become more pronounced in studies of other features of the pallial cavity. For example, Reynolds and Eernisse ([28], p. 94) stated that the lateral sense organs were described by Burne [59] but this is incorrect. Haszprunar [17] cited the lateral organs as being described by Thiele [24]; this paper was published earlier but refers to a putative lateral sense organ in gastropods. Small errors in interpretation, and their compounding effects, have dogged the study of molluscan sensory systems for over a century [50].

### Osphradium

The osphradium is a chemosensory structure found in most molluscan groups [18]. Within Polyplacophora, the osphradium *sensu stricto* is a pigmented sensory stripe in the roof of the pallial cavity between the anus and the first (posterior-most) gill ([19], p. 427). The osphradium in Polyplacophora is restricted to the order Chitonida, absent from all Lepidopleurida and possibly also from *Callochiton*[5], ES and JDS, pers. obs.], but homology with other molluscan osphradia urgently requires further investigation. It has been suggested that the osphradium is suppressed in Lepidopleurida because the gills multiply posteriorly [17,20,25]. Historically, its interpretation has been confused with the “branchial sense organs”, pigmented regions on the individual gills (see below). The histology of the osphradial epithelium was first described in several species by Haszprunar [17], one hundred years after it was first noted as a feature of chitons. Here, we have reviewed this history with a view to making robust comparisons among homologous features in the pallial cavity, and with the Schwabe organ.

Spengel ([60], p. 356) was the first to report an osphradium in chitons, which he described as a region of brownish pigment on the base of the gill, above the lateral and dorsal nerves. He found similar structures in other molluscs which correspond to the osphradium and concluded that they were olfactory sense organs, but he could not confirm this in chitons; that is, he described the same diffuse patch of pigment, at the base of the gills, in several different molluscs, and noted their collective homology but declined to use the word “osphradium” for Polyplacophora ([60], p. 381). If this structure in chitons represented a true osphradium, this would support homology between chiton gills and ctenidia of vetigastropods such as *Fissurella* and *Haliotis* (a conclusion Spengel was apparently not willing to commit to). Haller ([61], p. 28) retorted that Spengel’s “pigmentation” was simply blood visible through the epithelium, and that the structure was in fact glandular and not sensory ([59], p. 4). However, Haller [61] was describing pigmentation in the gill epithelium, not the pigmentation at the base of the gill to which Spengel [60] was referring. In fact, all of these refer to pigmentation on the individual gills, which we now regard as the “branchial organs” rather than the osphradium (see below).

Pelseneer ([38], p. 13) identified the “osphradium” as a papilla behind the gills ([38], p. 14, fig. 39) but further described it as forming a protective shield over a sensory area, apparently meaning the dorsal side of the ventral pallial lappet. He claimed this was comparable to the osphradium in other molluscs and is present only in chitons where gill rows do not extend to the anus (with the possible exception of *Callochiton laevis*, which he illustrated without his osphradium; ([38], fig. 8]). However, the figures in Pelseneer ([38], pl. 1, pl. 2) also appear to indicate the posterior pallial lappet as the “osphradium”, including a possible osphradium in *Leptochiton benthus* ([38], pl. 2, fig. 11), which he specifically compared to *Plaxiphorella tentaculifera* (not illustrated). Pelseneer [38] also indicated that the same type of papilla is visible on the anterior girdle in *Boreochiton marginatus*, but Nierstrasz ([50], p. 372) had already highlighted this misinterpretation. The osphradium as described by Pelseneer [38] is not the osphradium *s.s.* in other Chitonida, his suggestion that there may be a posterior osphradium in *Leptochiton* was therefore spurious. We suspect this may have been in error originally as the text citation in Pelseneer’s publication is to Figure 8 but *L. benthus* is actually illustrated ([38], fig. 11) The region (dorsal side of the ventral posterior pallial lappet) identified by Pelseneer [38] has not been described as a sensory epithelium by any other authors.

Arey and Crozier ([46], p. 252) noted that the “osphradium” is connected to the lateral nerve cord, and postulated that it was used for testing water quality, coordinating releasing of gametes or the coordination of gill movement and thus respiration. However, they were actually referring to Pelseneer’s [38] interpretation of sensory epithelium on the dorsal face of the ventral girdle lappet as the “osphradium” – thus, the osphradium would have been in the inhalant water current and well-positioned for these roles. They do also indicate the presence of a “ridge” posterior to the gills in *Chiton tuberculatus*, though this detail of the illustration is not referenced in the text [46]. The osphradium *sensu* Plate [19] was also described in *Ischnochiton herdmani* ([25], p. 598, fig. 5), and *I. aequigranulatus* ([25], p. 610, pl. 32, fig. 55). Yonge ([20], p. 384) also correctly described osphradia *s.s.* in Chitonida as “elongated structures lying along the roof of the pallial groove and extending from the anus as far as the post-renal gills”. This review standardised the interpretation of the osphradium but later authors’ references to original literature have repeatedly reintroduced the early conflation of the branchial organs and osphradia. Salvini-Plawen ([21] p. 250–251) does not describe the structure of the osphradium in much detail except to say that they are paired posterior terminal sense organs (p. 250–251). He also [21] considered the branchial organs in Lepidopleurida to be homologous to the osphradia in Chitonida, Gastropoda, Bivalvia, and terminal sense organs in Caudofoveata and Solengastres ([21], fig. 15).

More recently, Haszprunar [17] examined the histology of osphradial structures in six species within Chitonida and confirmed its absence in Lepidopleurida by examining *Lepidopleurus cajetanus*. Haszprunar [17] also reviewed historical suggestions as to the function of molluscan osphradium in general and postulated that it might be a chemoreceptor with a role in the reproductive biology of gonochoristic spawning molluscs, including Polyplacophora. Kamardin [62,63] did not describe the ultrastructure of the osphradium in any taxa, but did propose its involvement in the homing behaviour of *Acanthopleura gemmata*.

Where present, the osphradium *s.s.* was consistently described as a raised stripe of pigment associated with specific cell characters of the epithelium punctuated by sensory cells [17]. However, there are dramatic differences between them in terms of both histology and position in the species examined. Whereas the osphradium is located in the dorsal pallial cavity roof, between the last gill and the anus in *Ischnochiton rissoi*, *Lepidochitona cinereus* and *Middendorffia caprearum*, it can be found as far anterior as the second-to-last gill (and not adjacent to the mucous tract) in *Chiton olivaceus* and *Chiton corallinus*, and was reported below the anus in *Acanthochiton communis*[17]. The histology of the osphradium in the Ischnochitonina remains fairly consistent throughout the five species Haszprunar [17] examined: supporting cells with large ovoid nuclei and dense pigment granules surround sensory cells with basal perikarya and processes which reach the surface of the epithelium and carry short-rooted cilia, all with a length of around 40 μm. However, in *A. communis* the “sensory epithelium” is very different, with two distinct zones: a mucous zone on the mantle side up to 70 μm in length, and a sensory groove comprising supporting cells similar to those in Ischnochitonina, and sensory cells with pigment granules, oval nuclei and paddle cilia, all around 20–30 μm tall.

The description of the osphradium *s.s.* in Chitonina (i.e. *Ischnochiton*, *Lepidochitona*, *Middendorfia*, and *Chiton* spp.) is sufficiently different to the available information for *Acanthochitona* that it may not be possible to confidently infer homology of that “sensory epithelium” with the osphradium of other chitons. Yet osphradia have been described for several species of Acanthochitonina (Table 2); this highlights the need for further ultrastructural investigations. The chiton osphradium *s.s.* is similar to the cellular arrangement of the sensory epithelia in the Schwabe organ, and the lateral organs of Lepidopleurida; the major differences are in position, density of pigment, close association of pigment with nerves, and that the pigment appears to be infusing the epithelium from within the mesoderm in the Schwabe organ region.

### Branchial organs

The branchial organs are areas of innervated epithelium at the base (on the distal side) of individual gills. Sense organs specifically on the gills have been described in two ways, which we recognise as two separate structures: branchial organs or “sensory strips”, a pigmented line of thickened epithelium on the gill axis [19,49,59], and, separately, structural branchial organs, which are sensory “bumps” at the base of the gill [19,22,59] (Figure 8). These two structures have been conflated in the historical literature and also confused with the osphradium.

Several species have a “hard narrow line” of pigment running longitudinally down each gill, corresponding to an elevated ridge of sensory epithelium above the efferent nerve cord ([59], p. 8) (Table 2; Figure 8). It was this structure specifically, first described from *Acanthochitona discrepans* (Chitonida) [23] that was later defined as a “branchial sense organ” [5]. Whether branchial organs represent discrete organs, and whether they are sensory, has been a matter of long argument. Haller [61] first described tracts or ridges of modified epithelium at the base of the individual gills, apparently similar to a structural-type branchial organ, the bump at the base of the gill (Figure 8), but he considered them to be glandular in function [61]. Blumrich [23] redescribed the same structure but mentioned that the glandular epithelium also occurred anterior to the gills, and within this glandular epithelium, he identified sensory knobs ([23], p. 460). Haller ([62], p. 34) believed this epithelium was glandular and not sensory at all. Thiele [65] also doubted Blumrich’s [23] interpretation of a sensory function, but Simroth ([66], p. 262) followed Blumrich that the modified epithelium at the base of the gills was an “osphradium”. Lang [67] commented on the confusion but did not express a specific opinion.

Burne [59] searched for but could not find an osphradium in *Hanleya hanleyi* (Lepidopleurida)—although he described “ganglionic swellings” in all but the six anterior-most gills, saying the ganglia gave the nerve within each gill a beaded appearance. He regarded these “ganglia” as potentially a true osphradium [59], but we now consider neural structures within the individual gills as branchial organs. Plate ([5], p. 89) described secondary olfactory epithelium on the gills in *L. medinae*, with the nerve at the base of each gill connecting to a distinct ganglion. He observed five gill ganglia and two distinct olfactory patches plus additional one or two less distinct patches which may actually have been lateral organs on the pallial cavity distal wall. Plate ([5], p. 82) described the posterior gills in *L. asellus* as carrying innervated sensory epithelium on their outer edge (which he referred to as an osphradium in this earlier part of his work, but not in the later, more detailed descriptions [68], and said that the nerve supplying this forms a distinct ganglion under the base of each gill: that is, both a pigmented-type branchial organ and a structural-type branchial organ. In a 7 mm specimen examined by Plate, there were branchial organs on both sides of gills 2–5, yet in a slightly larger specimen (10 mm), gills 1 and 6 also had the same epithelium [5], so this may be correlated with development with age or simply an artefact of preservation.

Structural-type branchial organs appear to represent a local swelling of the epithelium at the point where branches from the lateral nerve cord enter the individual gills (Figure 8) and potentially also the efferent nerve penetrating the longitudinal axis of the gills. This structural type of branchial organ has been described in a member of Chitonida only from newly-settled post-larvae of *Acanthochitona “discrepans”* (probably *A. fascicularis*) which showed a sensory epithelium on the distal side of posterior larval gills, which was considered homologous to the osphradium in adults of the same species [69]. (As discussed above, there is potentially some doubt about the homology of the osphradium *s.s.* and that structure in *Acanthochitona*). Haszprunar [22] examined the branchial organ in *Lepidopleurus cajetanus*, and first referred to it as an osphradium, but in the final publication of his findings, reconsidered the structure and decided it was “not an organ” [17]. Several of the descriptions in the above literature, referring to putative osphradia in the Chitonida, better fit the profile of a pigmented-type branchial organ (on the gill, rather than at the base of the gill) than the histologically defined osphradium described by Haszprunar [17]. The position of serialised sense organs at the base of the gills would support an argument for a metamerised positional homolog of the osphradium in other molluscan classes. This interpretation would be contentious as there is no convincing evidence for neurological metamerism in Polyplacophora [51].

Plate [68] mentioned ganglia under the gills (i.e. structural branchial organs) but did not mention an olfactory epithelium, and also never compared his observations with those of Burne [59]. Yonge ([20], p. 385) claimed that Plate [5] regarded the branchial organs as a secondary structure, but this is not a wholly accurate representation of Plate’s published opinion. After reviewing published descriptions, Yonge [20] explicitly suggested that the lateral sense organs (see above) and branchial organs (not distinguishing the two types we have identified) collectively replaced the function of the osphradium in Lepidopleurida. This idea was echoed in later works [17,21,22].

The frequent association of pigment on the gills may be indicative of sensory epithelium similar in structure to that known from osphradia. Yet both types of branchial organs are structurally and positionally distinct and are not osphradia. The majority of descriptions of branchial organs refer to Lepidopleurida; however, many branchial organs (or, pigmented sensory epithelia on the gills) in Chitonida may have been initially described as “osphradia”, and these are clearly present in both clades.

### Anterior sense organs

Blumrich ([23], p. 463) reported seeing anterior “*paraneurale Epithelwülste*” (sensory epithelium) in *Lepidopleurus cajetanus* but the description does not precisely indicate the region where the epithelium starts (only that it was far in front of the gill row). Plate ([19], p. 425 *fide* von Knorre) refuted this. Thiele ([70], p. 395), who examined *Callochiton septemvalvis* (reported as *Chiton rubicundus*, [55], pointed out that he found the epithelium anterior to the gills to be elevated and that it seemed to be a sensory organ. Pelseneer [38] described that in the anterior region of the pallial cavity, adjacent to the foot (i.e. posterior of the mouth and mouth lappets) the lateral nerve cord enters the epithelium in “*Boreochiton*”, but the exact region is unclear: it is labelled as anterior of the holobranchial gill row but illustrated as containing nephridial tissue ([38], p. 14, fig. 26). Generally, we remain hesitant to make extensive use of the interpretations of Pelseneer [38] on this subject.

According to von Knorre [25] there are “anterior sense organs” in *Ischnochiton herdmani* (now *Stenoplax alata*) and *I. aequigranulatus* (now *I. bouryi*)*,* which he directly compared to the feature described by Blumrich [23] for “*Chiton” laevis* and *L. cajetanus* ([25], p. 599). He also clearly indicated the location of a pigmented olfactory organ, lateral to the first 5 gills and extending anteriorly to the mouth region in *Stenoplax alata*, with innervation from the lateral nerve cord. Yonge [20] also confirmed the occurrence of these “anterior olfactory organs” in *Lepidochitona cinereus*, but said they were absent in *L. asellus*. He also erroneously said they are innervated by the ventral nerve cord ([20], p. 384). Hyman ([49], p. 97) included the anterior olfactory organ in her review, describing it as an “anterior longitudinal sensory strip extending in the groove from the foremost gills to the side of the head”, but referring to Blumrich [23] and von Knorre [25] and no other reports.

We have visually inspected the surface epithelium in preserved specimens of *Ischnochiton* and *Stenoplax* for evidence of anterior pallial pigmentation and were not able to find anything in: *Ischnochiton rissoi, I. bouryi, I. erythronotus, I. hakodatensis, I. elizabethensis, Stenoplax alata,* or *S. purpurescens.* In one specimen of *S. purpurescens* (ZSM Mol 20090092) we found an area of potential pigmentation ventral to valves II and III. However this does not resemble the Schwabe organ as the pigment is more distal, directly under the valve apophyses, very diffuse over the pallial cavity roof and not forming a discrete patch; the epithelium also appears thinner in this region. The stripes as described by previous authors are oriented longitudinally in the mantle cavity [23,25], whereas the potential pigmented stripes we observed are transverse to the body axis. We suspect this is either an artefact or further evidence of extensive pigmentation throughout the pallial cavity in many species, but not homologous with the Schwabe organ.

## Conclusions

Previous investigations of sensory structures within the pallial cavity have been motivated by a search for a plausible osphradium homologue in chitons, and especially in Lepidopleurida. This has led to a situation where four different identified structures have been called the “osphradium” in primary anatomical literature: the pallial flap of Pelseneer [38], structural-type branchial organs (a lump at the base of the gill) [19], pigmented-type branchial organs (pigmented patches or stripes along the gill axis) [59], and the osphradium *sensu stricto* (raised pigmented epithelium between the posteriormost gill or gills and the anus) [17,19]. In addition to the osphradium, we recognise the lateral sense organs, which can be described as small, flat pads of cells rising above the surrounding epithelium and occurring along a longitudinal axis along the lateral edge of the pallial cavity wall. Branchial “sense organs”, in two forms, are further distinctly different to the polyplacophoran osphradium *s.s*. Whether the osphradium *s.s.* (as we have considered it here) in Chitonida is in fact homologous to other molluscan osphradia is not wholly clear.

Tomographic visualisation of the anterior nervous system of lepidopleuran chitons reveals a strikingly large and well-developed neural mass (brain) that is at odds with previous reports for the neuroanatomy of the group [4,5,59]. A fundamental assumption that chitons are “primitive” among Mollusca may have biased the interpretation of anatomical results by early, and even some contemporary researchers. Interestingly, however, there may also be considerable variation among species or between the major clades of Polyplacophora.

The anterior commissure is large, and well organised, and presents some properties of ganglionisation in having a large core of neuropil with all of the nuclei forming a thick distal layer. The various minor flexures within the commissure are not differentiated into identifiable ganglia, yet chitons are perhaps more organised than the generally accepted “incipient” cephalisation [46]. We also described the presence of a novel and distinct anterior sense organ, the Schwabe organ, in the more “primitive” living clade of chitons. However, the lateral nerve cord and not the anterior commissure innervate all sensory structures in the pallial cavity.

Unlike the elusive chiton osphradium, the Schwabe organ is distinctive and easily recognisable. It forms a novel anatomical synapomorphy for Lepidopleurida, and the variation in pigmentation will be taxonomically useful within the group. The Schwabe organ is elusive in that it lacks clearly differentiated tissues, ganglia, or other histological structures, yet it is easily recognisable in the living animals, is most likely a sensory structure, and evidently forms an important feature of Lepidopleurida, the earliest-diverging clade of living chitons. This study further demonstrates that suitable histological, tomographic and ultrastructural methods will continue to uncover truly surprising and novel discoveries for molluscan comparative anatomy, even in adult, macroscopic specimens.

## Materials and methods

Specimens used in this study were drawn primarily from the collections of the Zoologische Staatssammlung, Bavarian State Collection of Zoology (ZSM, Munich) as well as the Muséum National d’Histoire Naturelle (MNHN, Paris). We have extensively surveyed specimen material within the order Lepidopleurida and other phylogenetically proximate (or putatively proximate) taxa for evidence of pigment patches (Table 1). Additional fresh material for histological studies was obtained for *Leptochiton asellus* (Gmelin, 1791) in the NE Atlantic, and *L. rugatus* (Carpenter MS, Dall, 1879) in the NE Pacific. Material of *L. asellus* was collected by dredging in Gullmar fjord (depth 30–40 m), Sweden, in September 2012. Specimens of *L. rugatus* were collected intertidally in July 2008 at “Chinese Cemetery” [48°24′21″N 123°19′16″W], Victoria, Vancouver Island, British Columbia, Canada. *Leptochiton* spp. typically live adhered to the underside of rocks submerged in coarse sand. Specimens collected for these experiments were kept in aquarium for five days to clear the gut of sediment. Material of *L. rugatus* was then fixed in 4% formalin in seawater (i.e. chemical formalin 40% aqueous solution, diluted 1:10 in seawater), and later transferred to 70% ethanol for transport and storage (ZSM Mol 20081033). Material of *L. asellus* was fixed in 4% glutaraldehyde in 0.1 M sodium cacodylate buffer (pH 7.4). To prevent curling, animals were tied to a flat surface with fine string during fixation.

In preparation for semi-thin sectioning all specimens were post-fixed in 1% osmium tetroxide and then decalcified in 2% EDTA (pH 7.2) over a period of 36 hours. This process and subsequent acetone dehydration series, embedding, and tomographic model reconstruction, were followed as described in Ruthensteiner [71].

Prior to embedding, samples were kept in diluted Epon epoxy resin mixture (1:1 with 100% acetone) overnight at room temperature, left open to allow the acetone to evaporate. They were then embedded in Epon with DPM-30 accelerator for a further 24 hours at 60°C, according to the manufacturer’s instructions (Sigma).

Samples were serially sectioned at a thickness of 1.5 μm using a diamond knife (HistoJumbo 8 mm, DiATOME, Switzerland) on an automated microtome (Leica RM2255) and stained using Richardson’s solution [72]. Digital images of these sections were recorded using an Olympus E-600 digital camera mounted on an Olympus BX41 light microscope at a magnification appropriate to maximise specimen visibility. For an overall model of the anterior nervous system in both species, every fourth section was recorded digitally throughout the head region. For an additional high-resolution model of the Schwabe organ in *L. asellus*, and for the branchial organ in *L. rugatus,* every section within the region of interest was included. Images were reduced and contrast-enhanced for reconstruction using Adobe Photoshop CS3 before import into AMIRA v.5.3.3 (FEI Visualisation Sciences Group). Using AMIRA, images were aligned into a single stack, and materials of interest were highlighted throughout the stack before surface rendering and smoothing to produce 3D tomographic models.

### Electron microscopy

In order to characterise the ultrastructure of the Schwabe organ region, tissue samples were dissected out of six specimens of *L. asellus*, and fixed and embedded as above. Ultrathin sections were taken at 60–70 nm using a ultradiamond knife (Drukker International, B.V.) on a PowerTome XL (RMC Products) and Reichert Ultracut E (Reichert-Jung) ultramicrotomes. Sections were collected on formvar coated copper grids and stained with uranyl acetate and lead citrate before visualisation on a FEI Morgagni and a FEI CM 100 transmission electron microscope operating at 80 kV. Measurements were taken using ImageJ 1.46r (NIH).

In cases where semi-thin sectioning overreached the area of interest, some semi-thin sections were re-mounted onto resin blocks and ultrathin sections were then taken from these. Remounting was achieved by placing a drop of epoxy resin and an empty resin block over the section of interest and allowing this to polymerise over 48 hours at 60°C. The slide was then placed alternately in water at 100°C, and liquid nitrogen at -196°C until the section detached from the slide. Ultrathin sections could then be taken as described above. In order to compare the epithelium in the area of interest to areas of the pallial cavity without pigmentation, semi-thin sections from the AMIRA model serial sectioning were also remounted and ultra-thin sections taken from these in the same way. These semi-thin sections came from anterior to the position of the Schwabe organ. Finally, in order to characterise the lateral organs in *L. asellus,* one further semi-thin section taken at the centre of a lateral organ was remounted and ultra-thin sections were taken from this area (Figure 9E).

Previous attempts to characterise the surface of the pallial cavity in *L. rugatus* via SEM were hampered by excess mucus obscuring the surface epithelium (JDS, unpub. obs.). To avoid this problem, selected live specimens of *L. asellus* were treated with 500 mM N-acetyl cysteine (Sigma-Aldrich) in MES buffer (500 mM MES, 10 mM sucrose, 90 mM sorbitol, all Sigma-Aldrich, pH titrated to 5.5 using NaOH) for 30 minutes on a gentle shaker table at room temperature to remove mucus prior to fixation [33] (Figure 9A, B). Other museum specimens of *L. asellus* (ZSM Mol 20080293) were prepared for SEM without mucus removal treatment (not illustrated). Samples were fixed and dehydrated as described above, and dried using hexamethyldisilazane (Sigma-Aldrich) or a BAL-TEC CPD030 critical point dryer, and sputter-coated with gold using a Polaron E5100 SEM Coating System before visualisation on a LEO 1430VP (Zeiss) or FEI Quanta 200 scanning electron microscope.

### Availability of supporting data

The data sets supporting the results of this article are included within the article and its additional files.

## Competing interests

The authors declare that they have no competing interests.

## Authors’ contributions

JDS designed the study, conducted fieldwork, contributed to laboratory work, and wrote the manuscript; LHSR assisted with fieldwork, performed laboratory work, and wrote sections of the manuscript; ES made original observations leading to the conception of the study, contributed to laboratory work and wrote sections of the manuscript; MH contributed to laboratory work and interpretation of data; GB contributed to laboratory work and interpretation of data; MS contributed to the design of the study, assisted with laboratory work and interpretation of data. All authors contributed editorial input in the preparation of the final manuscript. All authors read and approved the final manuscript.

## Supplementary Material

Additional file 1: Figure S1 Tomographic model of the anterior nervous system in *L. asellus*. Ventral view with outline of body shown. Pink, nerve tissue; green, buccal nerves; yellow, Schwabe organs. The interactive 3D model can be accessed by clicking into the figure (Adobe Reader Version 7 or higher). Rotate model by dragging with left mouse button pressed, shift model: same action + ctrl, zoom: use mouse wheel (or change default action for left mouse button). Select or deselect components in the model tree or switch between prefab views. Click here for file

Additional file 2: Figure S2 Tomographic model of the anterior nervous system in *L. rugatus*. Ventral view with outline of body shown. Pink, nerve tissue; green, buccal nerves; yellow, Schwabe organs. The interactive 3D model can be accessed by clicking into the figure (Adobe Reader Version 7 or higher). Rotate model by dragging with left mouse button pressed, shift model: same action + ctrl, zoom: use mouse wheel (or change default action for left mouse button). Select or deselect components in the model tree or switch between prefab views. Click here for file
