# Supplementary material for: A new sensory organ in “primitive” molluscs (Polyplacophora: Lepidopleurida), and its context in the nervous system of chitons
Source: Front Zool. 2014 Jan 21;11:7. doi: 10.1186/1742-9994-11-7 (PMC3916795; doi:10.1186/1742-9994-11-7)
Supplement: Additional file 1: Figure S1 — Tomographic model of the anterior nervous system in L. asellus. Ventral view with outline of body shown. Pink, nerve tissue; green, buccal nerves; yellow, Schwabe organs. The interactive 3D model can be accessed by clicking into the figure (Adobe Reader Version 7 or higher). Rotate model by dragging with left mouse button pressed, shift model: same action + ctrl, zoom: use mouse wheel (or change default action for left mouse button). Select or deselect components in the model tree or switch between prefab views. [file 1742-9994-11-7-S1.pdf]

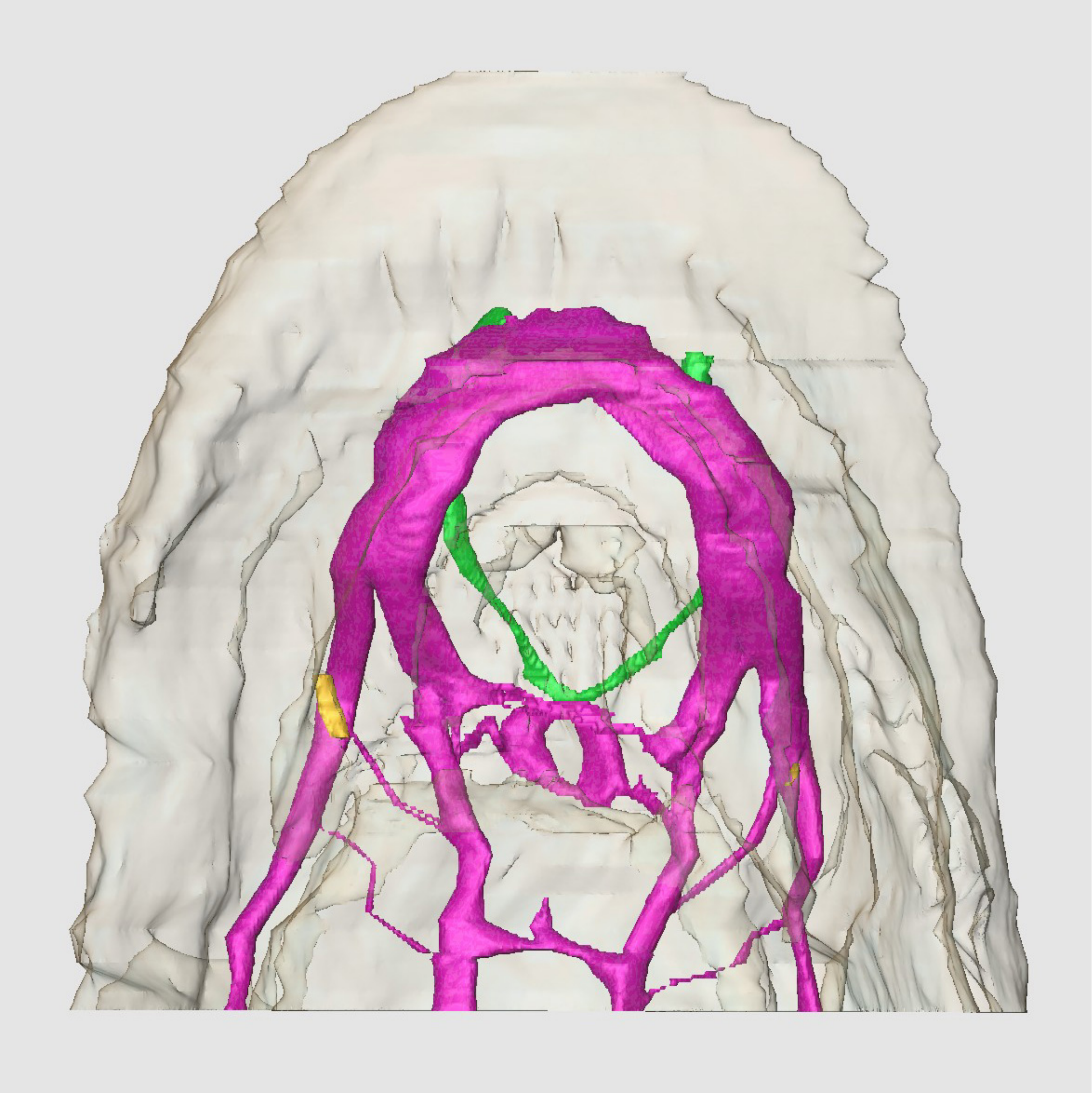

Supplementary figure 1 – Tomographic model of the anterior nervous system in *L. asellus*.

Ventral view with outline of body shown. Pink, nerve tissue; green, buccal nerves; yellow, Schwabe organs. The **interactive 3D model** can be accessed by clicking into the figure (Adobe Reader Version 7 or higher). Rotate model by dragging with left mouse button pressed, shift model: same action + ctrl, zoom: use mouse wheel (or change default action for left mouse button). Select or deselect components in the model tree or switch between prefab views.
